# Development of a Respiratory Inductive Plethysmography Module Supporting Multiple Sensors for Wearable Systems

**DOI:** 10.3390/s121013167

**Published:** 2012-09-27

**Authors:** Zhengbo Zhang, Jiewen Zheng, Hao Wu, Weidong Wang, Buqing Wang, Hongyun Liu

**Affiliations:** 1 Department of Biomedical Engineering, Chinese PLA (People's Liberation Army) General Hospital, 28 Fuxing Road, Haidian District, Beijing 100853, China; E-Mails: zhengbo_zhang@hotmail.com (Z.Z.); wmdwh@126.com (H.W.); wangbuqing040625@126.com (B.W.); ylooliu@163.com (H.L.); 2 The Quartermaster Research Institute of the General Logistic Department, 69 Lumicang Hutong, Dongcheng District, Beijing 100010, China; E-Mail: zhengjiewen100@yahoo.com.cn

**Keywords:** respiratory inductive plethysmography, pulse amplitude modulation, time-division multiplexing scheme, LC oscillator, sensing unit, multiple sensors, wearable system

## Abstract

In this paper, we present an RIP module with the features of supporting multiple inductive sensors, no variable frequency LC oscillator, low power consumption, and automatic gain adjustment for each channel. Based on the method of inductance measurement without using a variable frequency LC oscillator, we further integrate pulse amplitude modulation and time division multiplexing scheme into a module to support multiple RIP sensors. All inductive sensors are excited by a high-frequency electric current periodically and momentarily, and the inductance of each sensor is measured during the time when the electric current is fed to it. To improve the amplitude response of the RIP sensors, we optimize the sensing unit with a matching capacitor parallel with each RIP sensor forming a frequency selection filter. Performance tests on the linearity of the output with cross-sectional area and the accuracy of respiratory volume estimation demonstrate good linearity and accurate lung volume estimation. Power consumption of this new RIP module with two sensors is very low. The performance of respiration measurement during movement is also evaluated. This RIP module is especially desirable for wearable systems with multiple RIP sensors for long-term respiration monitoring.

## 1. Introduction

Breathing is an important physiological function, and long-term monitoring of respiratory function is desirable for the diagnosis of a variety of diseases. Several techniques are available for wearable respiratory monitoring, such as electric impedance plethysmography (EIP), respiratory inductive plethysmography (RIP), magnetometers/strain gauge sensors, and piezoresistive materials displacement sensor. Compared with other techniques, RIP has the advantages of greater accuracy, better sensitivity and higher safety for patients, and has been used widely in clinical and research settings [1–3]. The new American Academy of Sleep Medicine (AASM) scoring guidelines recommends RIP technology as a more reliable way to measure respiratory effort in AASM accredited sleep centers [4]. ‘LifeShirt’, a once well-known wearable system, integrated RIP technology in a T-shirt for non-invasive assessment of the breathing patterns [5]. Many reports on clinical experiments using ‘LifeShirt’ and validation test on this wearable cardiopulmonary monitoring system can be found [6,7].

RIP was firstly introduced as a non-invasive respiratory monitoring device in 1977 by Cohn, with two winding coils of wire within elastic bands encircling the rib cage (RC) and abdomen (AB) [8]. The inductance of the conductive loop is a measure of the cross sectional area encircled and the change in self-inductance reflects the respiration of the patient. The inductance measurement circuitry includes a variable-frequency LC oscillator with the coils as the inductive element, measuring electrical frequency or amplitude of the oscillator to determine the inductance. Several possible circuit implementations of this technology can be found in the US patent literature [9,10]. Cohen [11] presented a detailed technical description of an RIP design by directly measuring the frequency of a Colpitts oscillator to determine the changes in cross sectional area that occur during breathing.

To achieve sufficient measurement accuracy and rate, the LC oscillator circuit containing the conductive coil must have a sufficient high Q factor. A plot of energy *versus* frequency for an oscillator will show a bell shaped curve centered at the resonant frequency. A high Q oscillator oscillates over a narrower band of frequency and hence is more stable. The stability of the oscillator frequency and hence the minimum frequency change that can be detected, is a direct function of the Q-factor. As the stability of the Colpitts oscillator itself is usually limited and a single-loop inductive sensor may lower the circuit Q value further, it is not easy to design such an oscillator with high stability. In some designs, small RF transformers with the transducer as the primary coil are used to improve the Q value and magnify the inductance of the transducer. High Q-factor inductive transducers by means of increasing the number of turns of the conductor were also developed to improve the sensitivity and stability of the RIP circuit [12,13]. The mutual inductance between RIP sensors is another problem for a system with multiple inductive sensors. Cohen's experiments have demonstrated that chest and abdominal band oscillator frequencies have to be sufficiently separated to decrease modulation and avoid frequency locking. Thus for a respiration monitoring system with multiple inductive sensors, designing a stable oscillator for each inductive sensor while keeping the frequency separation between each other is very challenging. For wearable respiratory monitoring systems powered by batteries, power consumption is a crucial factor to be considered, and small size of the printed circuit board (PCB) is also desirable for portable use. Conventional RIP design using an LC oscillator increases the chip count and complexity of power management if multiple inductive sensors are required.

Sinton [14] described an RIP employing conventional EIP to sense the coil's self-inductance, with the advantage of being compatible with existing EIP systems. With a constant current from the EIP flowing through the RC and AB RIP sensors, this method does not need an LC oscillator for inductance measurement. In Sinton's design, special inductive sensors with ten-turns of copper wire were fabricated to acquire enough sensitivity with the existing EIP systems. If a conventional RIP sensor with one single-loop wire was used in this method, a constant current source with more than ten times of output current would be needed to excite the inductance to generate enough amplitude response. In this situation, continuously large current flowing through sensors would increase the power consumption dramatically.

We propose a method of pulse amplitude modulation (PAM) for indirect inductance measurement without using a variable-frequency oscillator [15,16]. For one RIP sensor, large current from a constant-current source is fed to it momentarily and periodically, and its inductance is measured during the pulse excitation period. Different from Sinton's design, the feeding current is not flowing through the inductive sensor continuously. Thus the power consumption is reduced dramatically with the technique of PAM, without losing sensitivity due to the momentarily large feeding current.

In this paper, we present an RIP module using PAM to reduce power consumption and time-division multiplexing scheme (TDM) to support multiple inductive sensors. Further, in order to increase the amplitude response of the sensor during PAM, we optimize the sensing unit. Each sensing unit consists of an inductive sensor with a variable inductor and a capacitor with constant matching capacitance. Such kinds of sensing units can generate a much larger magnitude response when excited by a certain high frequency current, compared with conventional inductive sensors. With the use of PAM and TDM, magnetic coupling due to the mutual inductance between multiple sensors can also be easily avoided. Control signals used by PAM and TDM are all generated by a micro-controller. Thus it becomes convenient to make changes on the control signals to support multiple RIP sensors without increasing the complexity of the circuit. Also, the application time of the feeding current for each sensor can also be modified automatically according to the amplitude response of the sensing unit to acquire the optimal gain and sensitivity for each sensor. This RIP module is especially desirable for wearable systems with multiple RIP sensors for long-term respiration monitoring. Performance on output linearity and the accuracy of respiratory volume estimation are tested. The abilities of respiratory events detection, respiration measurement during movement, and cardiogenic oscillation capturing are also evaluated.

## 2. Experimental Section

### 2.1. Principle of Measurement

**Figure 1.**
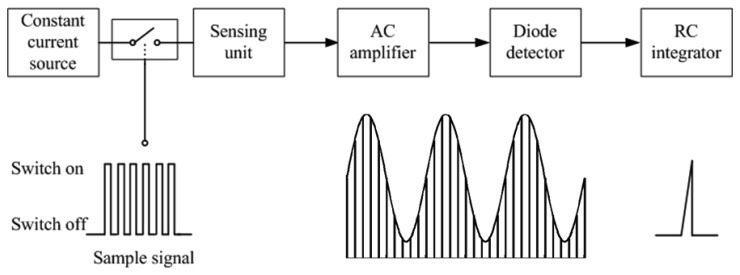
Block diagram of how PAM works in the RIP module.

Figure 1 illustrates how the PAM technique works in the RIP module. A constant-current source generates a peak-to-peak sinusoidal current of 15 mA at 400 kHz. The constant current is fed to each inductive sensor (sensing unit) briefly, 100 μs for example, through a CMOS analog switch controlled by a digital gating signal from the MCU. Change in self-inductance of the inductive wire caused by breathing movement results in change of AC impedance of the sensing unit. Thus the voltage across the sensing unit can be detected to reflect the respiratory movement. The analog signal from the sensing unit is amplified and demodulated within 100 μs, and the output is sampled at the end of the excitation. As breathing is a relatively low frequency signal, the frequency of PAM is set to 50 Hz. Thus the sensing unit is connected to the constant-current source every 20 ms, and the pulse width of the pulsed excitation current to each sensor is 100 μs. Although the pulsed excitation current to the sensing unit is large, its average current is actually only 1/200 of 15 mA because of the very low duty cycle. In a preferred embodiment, an RC integrator is used to remove the high frequency noise and improve the signal-to-noise ratio (SNR) further, as shown in Figure 1.

**Figure 2.**
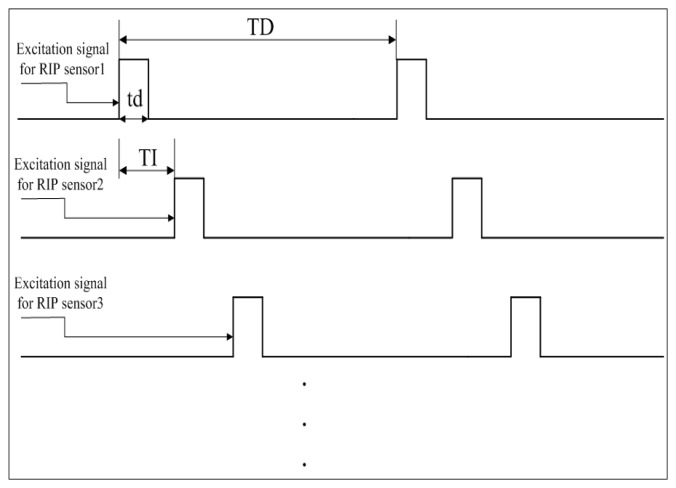
Description of how TDM works in the RIP module.

Based on PAM, the TDM technique is further implemented to support multiple RIP sensors. Figure 2 illustrates how TDM works. All the RIP sensing units are excited one-by-one by the same current source, and at each time there is only one sensing unit connected with the current source. As shown in Figure 2, the first RIP sensor is excited in the application time of ‘td’, then the second RIP sensor will be excited after time of ‘TI’. For each RIP sensor, the application time is the same as ‘td’, and the excitation period is the same as ‘TD’. Thus the sampling rate for each sensing unit is [1/TD] and the number of RIP sensors that can be supported by this module theoretically is [TD/TI]. Because there's only one RIP sensor working at any time, this method can avoid the cross-talk between RIP sensors effectively. As the impulse response of the sensing unit is approximately 40 μs (see below the section on the sensing unit), the width of TI should be at least 40 μs longer than that of ‘td’.

For multiple RIP systems, the approach of combining PAM with TDM can further reduce the power consumption of the whole system, without increasing the signal conditioning circuits by multiplexing for all sensing units. Thus the low power consumption and compact size of PCB can be guaranteed. In our RIP module, the constant-current source and signal conditioning circuits including AC amplifier, demodulator, and RC integrator are multiplexing for all sensing units.

### 2.2. Sensing Unit

As the average inductance for a conventional RIP sensor, made with one single-loop wire, is about 2∼3 μH, the AC impedance of the inductive belt itself is usually less than 10 Ω when it is excited by a sinusoidal current at 400 kHz. The AC impedance can be calculated by Equation (1), where *L* represents the inductance of the sensor, *f* represents the excitation frequency, and *Z* represents the AC impedance:

(1) [Z=|j∗ω∗L|=|j∗2∗π∗f∗L|]

**Figure 3.**
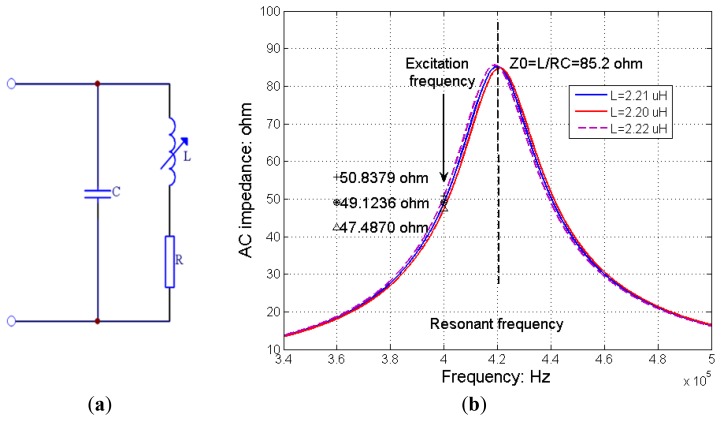
The structure of a parallel LC sensing unit and the impedance-frequency relationship of the transducer with variable inductance. (**a**) A parallel LC sensing unit; (**b**) Transfer function of the LC sensing unit.

If the excitation current is not large enough, limited to the gain-bandwidth product of the operational amplifier, an amplifier with very high gain-bandwidth product or multiple wide-band amplifiers would be required to get enough gain before the respiration waveform can be effectively demodulated by a diode detector circuit. In order to improve the amplitude response of the inductive sensor, we redesigned the sensing unit. As shown in Figure 3(a), the sensing unit consists of a variable inductor (L) and a constant capacitor (C) forming a parallel LC circuit. Here L represents the inductance of the RIP sensor, R represents the DC resistance of the wire (the self capacitance of the wire is omitted in this illustration), and C represents a matching capacitor with a proper capacitance to set the central frequency of the LC circuit. Compared with conventional inductive sensors without a matching capacitor, this design can improve the AC impedance of the sensing unit when the sensing unit is excited by a high frequency current around its resonant frequency. Thus one wide-band amplifier is enough to generate desired gain and enough output amplitude for effective signal demodulation. Compared with a conventional RIP sensor, this sensing unit is actually a frequency selection filter. As shown in Figure 3(b), the parallel LC sensing unit forms a band-pass filter with a variable central frequency. The output amplitude of the filter will change according to the shift of the central frequency caused by the changes of self-inductance during breathing. To keep a monotonic relationship between the change of AC impedance and that of the self-inductance during inductance measurement, the frequency of the excitation current should be set apart from the central frequency of the LC circuit. In Figure 3(b) for example, the excitation frequency is set at the left side of the LC resonant curve with a frequency separation of 20 kHz from the resonant frequency. The resonant frequency of LC sensing unit is nearly 420 kHz with L = 2.21 μH, C = 65 nF, R = 0.4 Ω, and the AC impedance of the sensing unit at resonant frequency is 85.2 Ω. The frequency of excitation signal is 400 kHz. We can see that the resonant frequency and AC impedance of the parallel LC circuit varies with the change of the inductance. ‘*’ represents the AC impedance of the sensing unit with L equal to 2.21 μH, ‘Δ’ represents the AC impedance with L equal to 2.20 μH, and ‘+’ represents the AC impedance with L equal to 2.22 μH. Our measurement shows that the shift of the central frequency for the sensing unit is less than 40 kHz with the encircled circumference of the inductive band changing from 70 cm to 110 cm, which covers most individual's thoracic and abdominal circumference, so 20 kHz frequency separation between the resonant frequency and the excitation frequency is enough to avoid polarity reversion during measurement and still maintains enough amplitude response and sensitivity.

In fact, the frequency shift caused by the change of self-inductance during natural breathing is so small that the relationship between the change of amplitude and that of self-inductance can be deemed as linear. For single-loop inductive sensor used for adult subjects, if the excitation frequency is set to 400 kHz and the central frequency of the sensing unit is set about 420 kHz, the AC impedance of each sensing unit is typically in the range of 30∼70 Ω at 400 kHz, depending on the size of the subject and the property the inductive sensor.

A commercial inductive belt can be used by this design, and the only additional item then required to produce a complete sensing unit is the selection of a matching capacitor. The capacitance should be accurately selected to make the sensing unit at a desirable resonant frequency field. A wide range of subject sizes (neonate, child, adult *etc.*) may be easily accommodated by correct selection of the matching capacitor with larger capacitance for smaller belts and smaller capacitance for larger belts.

### 2.3. Transient Response of the Sensing Unit

As the high-frequency excitation current is fed to each sensing unit momentarily and periodically, the transient response of the sensing unit is a key factor to be considered in designing such an RIP module using the techniques of PAM and DTM. The rise time of the transient response determines the duration time of ‘td’ and ‘TI’ (the definition of ‘td’ and ‘TI’ see Figure 2). For a self-made RIP sensor with one single-loop wire encapsulated in an elastic band, our measurement shows that it takes approximately 40 μs for the transient response to reach a steady state when the sensing unit is excited by a 400 kHz constant sinusoidal current. Thus the pulsed width (‘td’) of the excitation current and the width (‘TI’) between successive excitation should be no less than 40 μs. When other type or commercial RIP sensor is used, rise time of the sensing unit might be different. It is easy to test and determine the ‘td’ and ‘TI’ by observing the rise time of the transient response. If the RIP module is not heavily equipped with large amount of inductive sensors, we can set ‘td’ and ‘TI’ to a relatively large width. Thus we don't need to test the rise time for each sensor.

### 2.4. System Block Diagram

**Figure 4.**
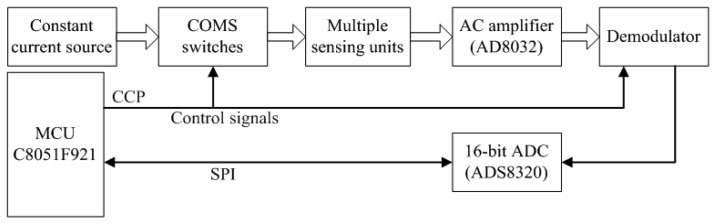
System block diagram of the RIP module.

Based on the principles described above, we designed the RIP module supporting multiple inductive sensors with micro-controller and integrated circuit chips. A low power MCU C8051F921 was used to generate control gating signals applied to the multiple COMS switch used by the techniques of PAM and TDM. A 16-bit ADC ADS8320 was used to sample the DC-coupled respiration signal at the end of each excitation for each sensing unit. For each sensing unit, during the application time, the voltage across the sensing unit is amplified by a wide-band amplifier AD8032 and then demodulated by a diode detector. To improve the SNR and remove the high frequency noise further, the diode detector is followed by an Op-amp RC integrator with a small time constant. As the time constant is small, the output voltage of the Op-amp RC integrator is shaped to a sharp saw-tooth wave during the application time ‘td’. For each sensing unit, the final output signal is sampled at the peak amplitude of each saw-tooth wave by ADS8320. All sampled digital signals are sent out by a universal asynchronous receiver/transmitter (UART). Figure 4 illustrates the system block diagram of the RIP module.

As the compare/capture/PWM (CCP) module is integrated into the MCU, it is convenient to generate the control signals used by PAM and TDM. Also as the application time of the ‘td’ can be modified automatically by the MCU, the amplitude of the saw-tooth wave generated by the RC integrator can be adjusted by changing the width of ‘td’. Large width of ‘td’ results in large output amplitude. With an embedded thresh-hold comparison algorithm running in the MCU, the output amplitude of all sensing units can be guaranteed relatively in a proper output range desirable for AD conversion. In our system, as the reference voltage for ADC is 3.3 V, the thresh-hold value is set to 2.5 V. This property of automatic gain setting is very important for multiple RIP sensors, as the AC impedance of each sensing unit might not be adjusted at the same level when they are excited by the same current source, even the matching capacitor is delicately selected.

### 2.5. Performance Test

**Figure 5.**
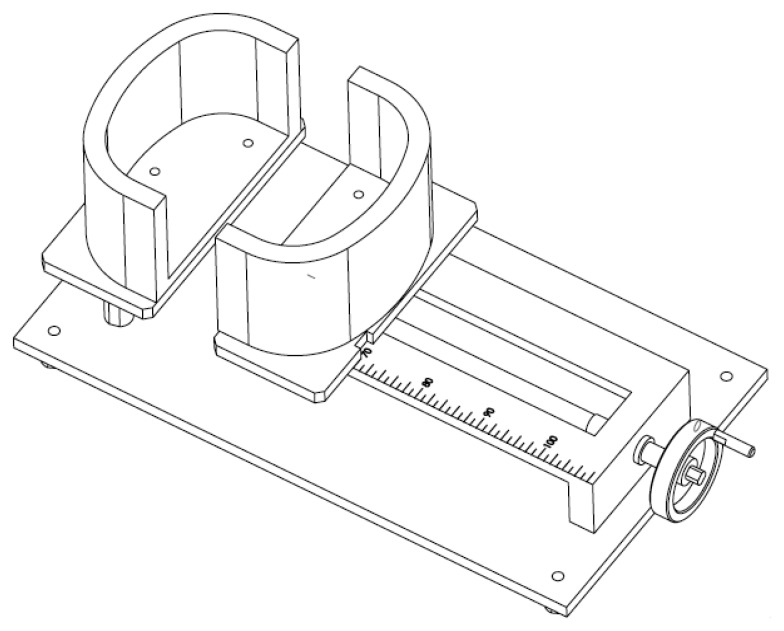
Illustration of the self-made apparatus for RIP performance test.

An important quality of inductance plethysmography is that the signal depicted is linear. To evaluate the proposed design, the linearity of RIP of this module was firstly tested. Though the module can support multiple sensors working at the same time, for the convenience of test, we only used one inductive belt for linearity test. The linearity of output with cross-sectional area was tested by a self-made apparatus with a changeable cross-sectional area similar to human beings (as shown in Figure 5). Turning the handle can change the distance between the two sections of the apparatus to simulate the change of the cross-sectional area of the chest. Through the ruler mark, the cross-sectional area and corresponding circumference can be easily calculated). The changing scope of cross-sectional area is from 360 cm^2^ to 760 cm^2^, with corresponding circumference changing from 70 cm to 110 cm. The cross-sectional area was changed manually and the corresponding output voltage from the RIP was measured and analyzed to test the linearity.

The second important quality of RIP sensor is that being capable of noninvasive ventilation measurement after calibration. We connected two inductive sensors for RC and abdomen (AB) respiration measurement and used the two-compartment respiratory system model by Konno and Mead [17] for calibration. As for quantitative measurement of respiratory volume, Qualitative Diagnostic Calibration (QDC) procedure [18] was used to calibrate the RIP with the FlowAnalyser™ PF-300 (Biomedequip, Strategic Medical Sales, Inc. Bedford Heights, OH, USA) as the volume reference. With calibration, volume signal can be derived by Equation (2), where *ΔVt* represents the change of tidal volume (Vt), *ΔRC* and *ΔAB* are the changes in RIP rib cage and abdomen signals, and K and M are calibration coefficients. The RIP volume signal is the weighted sum of the RC and AB inductive belt output voltage. PF-300 volume signal is the numerical integral of the corresponding flow signal. PF-300 supports bidirectional flow measurement with temperature compensation. With FlowLab™ software package, accurate flow measurement within a low flow range of −20 to 20 sL/min and a high flow range of −300 to 300 sL/min with an accuracy of ±1.75% of reading can be achieved. Fifteen volunteers took part in the test in supine position and each test lasted 30 minutes. Breath-by-breath Vt estimated by RIP was compared with the volume calculated from the PF-300. The absolute percentage error of breath-by-breath Vt between two systems was calculated. Average Vt of each subject from two systems was also computed respectively. Correlation coefficient and the paired T test on average Vt between calibrated RIP and PF-300 were presented;

(2) [ΔVt=K∗ΔRC+M∗ΔAB]

At the final stage of each respiratory volume test, stimulated airway obstruction was also performed. The subject simulated airway obstruction by closing his mouth and attempting to take in air by moving his abdomen in and out. RIP calibrated volume with the QDC coefficients was observed to produce minimal output during simulated airway obstruction.

The power consumption is an important factor for wearable systems powered by battery. The power consumption of the RIP module with two inductive belts was tested using digital multimeter 34401A (Agilent Technologies, with 6.5 digits of resolution). The 34401A has four DC current ranges: 10 mA, 100 mA, 1 A, and 3 A. The DC current can be tested with a high accuracy of ± (0.01% reading + 0.004% range).

Motion artifact is a major limitation in most practical implementations of wearable health monitoring devices. Biomedical signals are sensitive to body movement. Moreover, the spectrum of motion artifact usually overlaps with physiological signals and changes in different movement conditions. We tested the performance of our RIP module for RC and AB respiration monitoring during movement. Two RIP sensors were integrated into an elastic T-shirt in the position of chest and abdomen, and connected to the RIP module. We also used a tri-axial accelerometer to objectively capture body movement, and its outputs were used as references for adaptive motion artifact cancellation. All these signals were sampled at 50 Hz.

Finally, the ability of capturing cardiogenic oscillations from respiration waveform recorded by RIP was also tested. One RIP belt was placed around the xiphoid position to capture respiration movement and cardiogenic oscillations during normal breathing and simulated holding breath. ECG signal was also recorded simultaneously.

## 3. Results and Discussion

### 3.1. Linearity of Output with Cross-Sectional Area

**Figure 6.**
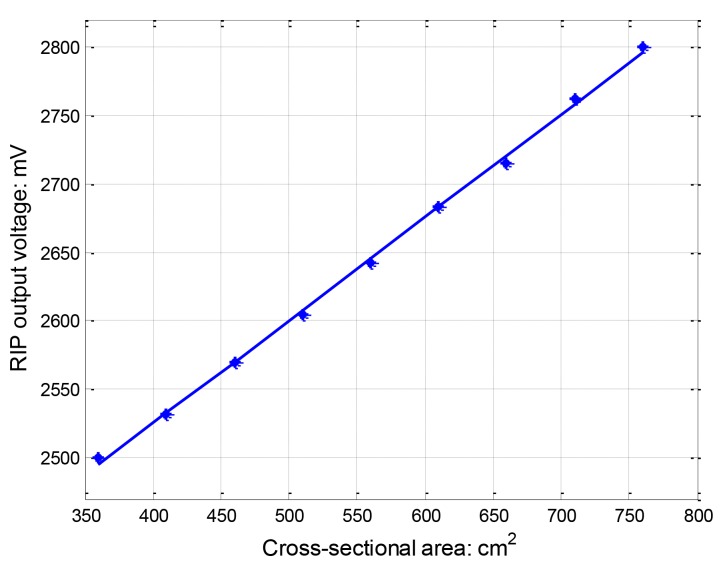
Relationship between RIP output voltage and coil cross-sectional area. Regression line is based on linear polynomial least-square fit.

Figure 6 depicts RIP output voltage *vs*. cross-sectional areas for the curved shape from the self-made testing apparatus. A linear polynomial least-square regression equation was fitted to the data. The first-order regression equation was *y* = 0.75*x* + 2223.7, where y is RIP output voltage (mV) and x is the cross-sectional area (cm^2^). The R-Square correlation coefficient between actual data and the output of a fitted curve is equal to 99.93%, which means that first-order linear equation fits the data very well. The linearity is comparable with other RIP for lung volume measurement reported [14,19].

Linearity is an important quality for RIP. Accordingly, output should change in proportion both when the band is stretched and relaxed. Figure 3(b) illustrates theoretically how the AC impedance of the sensing unit varies throughout the operating range of stretch. For typical physiological measurements, the operating range of relative stretch is from 0.1% or less up to 10∼15% or less for most physiological process. Linville [13] measured seven different RIP transducers to compare the relative changes in inductance per stretch. The largest change in inductance is only 0.149 μH at the maximum stretch of 186%, with belt length elongating from 72.1 cm to 134.1 cm. Thus for most physiological process, variation in inductance is small enough to generate a linear relationship between changes in AC impedance and the stretch. In Figure 3(b), the deviation from linearity is only 4.6% with the relative change of ±0.01 μH around 2.21 μH (0.45%). Experimentally testing shows (Figure 6) there is a good linear relationship between the cross-sectional area and the output.

Actually, the linearity of the RIP is determined by both the circuits and the structure of the sensor. In this paper, a simple, self-made sensor with single-loop and sinusoid coil of wire insulated and placed within an elastic band was used for linearity test. This kind of structure is most widely used in the RIP sensor design. As a result, for ordinary type of RIP sensors, this module is expected to generate a good linearity with change of the across-sectional area.

Another important characteristic of the sensing unit is that the slope of the transfer characteristic in the operating range of the relative stretch should be as great as possible. With larger slopes, relative stretch can be inferred from measured AC impedance characteristic with more accuracy. Theoretically, slope of the transfer characteristic for our sensing unit is a function of Q value of the LC circuit, which is mainly determined by the inductance of the sensing belt. Sensing unit with larger Q values is preferred to generate steep response characteristics while maintaining possible linear zone when the sensor element varies with stretch. The AC impedance of the sensing unit is determined by both the Q factor and the excitation frequency. From the transfer function illustrated in Figure 3(b), we can see the excitation frequency should be located in the relatively linear area, and should not be too far away from the resonant frequency to maintain a large AC impedance. Also, the excitation frequency should not be set too near to the resonant frequency to avoid polarity reversion during measurement. In our design, frequency separation between the resonant frequency of the LC sensing unit and the excitation frequency is set to 20 kHz. For different usage purposes and different type of inductive sensors, the frequency separation might be different.

### 3.2. Accuracy of Respiratory Volume Estimation

Table 1 shows the results of average Vt and standard derivation (SD) of the two systems. For each test, absolute percentage error of average Vt between two systems was also calculated, and calibration coefficients of RC (K) and AB (M) by QDC procedure were also presented. For breath-by-breath Vt comparison, sequential Vt collected from our RIP and PF-300 were aligned in adjacent columns of a data spreadsheet for each participant for evaluation. Of a total of 11,437 validation breaths, 93.85% (10,734 breaths) of the breath-by-breath Vt measured by our RIP have been shown to be within ±10% of simultaneous PF-300 measurements, 98.10% (11,220 breaths) within 15%, and 99.03% (11,326 breaths) within ±20%. The correlation coefficient of average Vt in 15 subjects between two systems was 0.9988 (P < 0.001, n = 15). The paired T test (P = 0.918, n = 15) demonstrated that there was no significant difference on average Vt measurement between two systems. Accurate ventilation measurement can be acquired by this new RIP after calibration under unchanged posture condition. The result is also comparable with other experiment [20].

**Figure 7.**
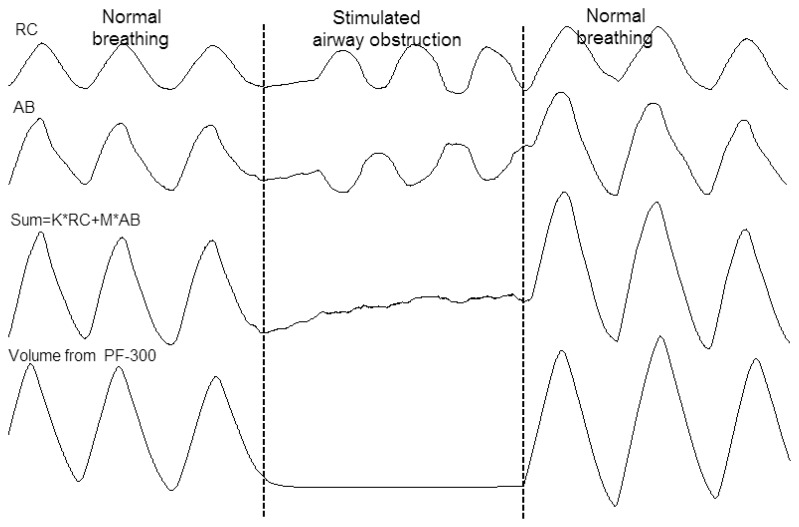
The weighted sum of RC and AB band signals during normal breathing and simulated airway obstruction.

Figure 7 shows the simultaneous recording of ventilation from PF-300 and respiration trace of RC and AB from RIP during normal breathing and simulated airway obstruction. The weighted sum output was formed by adding the RC and AB signal multiplied by its volume/motion coefficients obtained from the QDC procedure. True paradoxical breathing can be observed during the stimulated airway obstruction, and the derived value of lung volume from our RIP values by thoracic and abdominal movements is consistent with the PF-300's volume value.

Non-invasive ventilation monitoring is desirable for ambulatory physiological monitoring systems, and accurate ventilation monitoring can offer detail information about respiratory disorder, such as sleep apnea, COPD, and asthma *etc.* Performance tests showed this RIP module can acquire accurate Vt measurement after QDC calibration. Some scholars have doubted the reliability of long-term ventilation monitoring by the calibrated RIP method, as the chest and abdominal contribution to ventilation may be different with changes of body posture [21], so for a wearable monitoring system with RIP sensors for ventilation estimation, one of the critical problems is how to keep the estimation of ventilation within an acceptable level when posture changes in real life. Posture-related calibration is a method which has been put forward recently, where acceleration sensor is used for posture identification [22]. In different posture, the system automatically chooses the corresponding calibration ratio of chest and abdomen for ventilation estimation. As the validation of ventilation monitoring during exercise by ‘LifeShirt’ system has been reported [23,24], the performance of our RIP module in ambulatory conditions needs to be tested further.

Though we have not yet conducted any experiments on sleep apnea monitoring and detection using this RIP module, the original type we developed had good performance in detecting and distinguishing obstructive and central sleep apnea [25]. Figure 7 indicates that this RIP module is capable of capturing obstructive breathing event with calibrated Vt and paradoxical RC and AB movement. The performance of this RIP module in sleep medicine can be tested in the future.

### 3.3. Respiration Measurement during Movement

**Figure 8.**
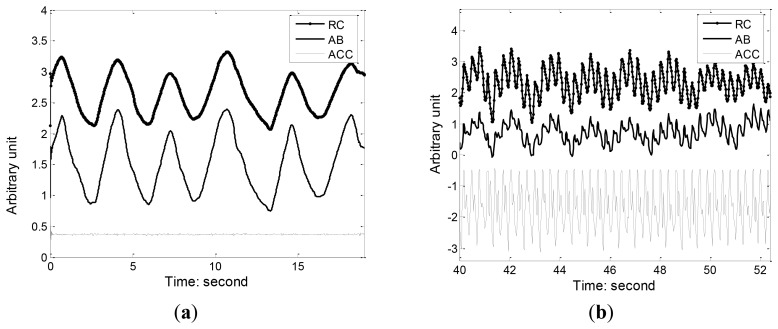
RC and AB respiration and accelerometer reference signal during standing and running. (**a**) respiration during standing; (**b**) respiration during running.

**Figure 9.**
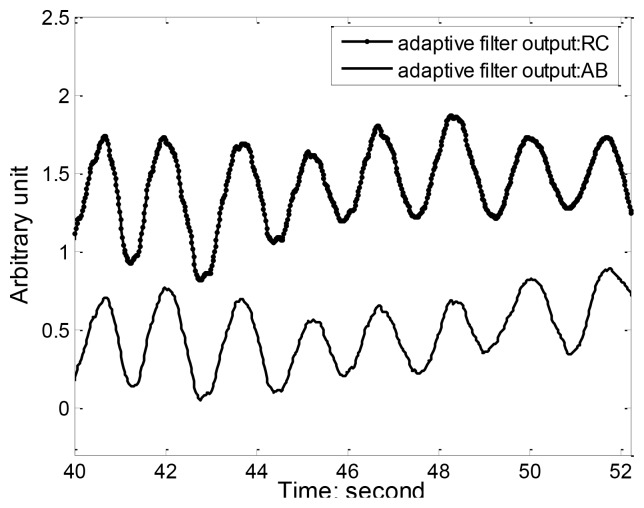
Output of adaptive filtering using least mean square (LMS) algorithm with accelerometer signal as reference.

Figure 8 illustrates the RC and AB respiration and accelerometer (ACC) reference signal during standing and running. We can see that running movement can insert a large amount of motion artifact and distortion into the respiration waveform, making it hard to detect the onsets and peaks of each breath. Since running is accompanied by toward accelerations that can be captured the ACC, we used the adaptive filtering to recover the respiration component from the movement contaminated RIP signal. Figure 9 shows the results of adaptive filtering using least mean square (LMS) algorithm. For LMS implementation, the length of the filter is 50, and the step size is 0.01. A moving average filter followed the LMS adaptive filter to make the respiration waveform cleaner. As ACC offers a practical and low cost method of objectively monitoring human movement, ACC has been widely integrated in most wearable systems and its outputs were used as references for adaptive filtering of motion artifact. Keenan DB [26] also demonstrated the application of wavelet and LMS adaptive filtering to improve the accuracy of respiratory measurement with ACC as reference signal.

### 3.4. Cardiogenic Oscillations during Breath-Holding

**Figure 10.**
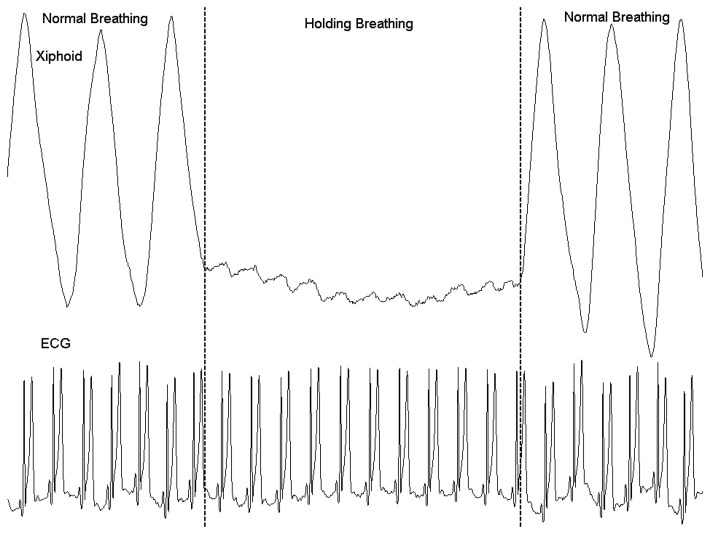
RIP band signals at the level of the xiphoid and ECG during normal breathing and simulated breath holding.

Figure 10 shows the xiphoid movement captured by RIP belt and simultaneous recording of ECG signal during normal breathing and simulated holding breath. Cardiogenic oscillations can be clearly observed during the breath holding stage, and they are synchronized with the ECG signals. Thoracocardiography (TCG), based on the principles of RIP, has been proposed as a non-invasive method for cardiac output measurement [27]. According to chest TCG, respiratory movements during natural breathing account for approximately 95% and left heart ventricle activity (cardiogenic oscillations) for 5% of the amplitude of the waveform recorded at the level of the xiphoid [28]. Our RIP can effectively capture the cardiogenic oscillations and can be used for research of non-invasive left ventricular stroke volume estimation by ECG triggered ensemble averaging and digital band-pass filtering [27].

RIP is the most frequently used, established and accurate plethysmography method to estimate lung volume from respiratory movements. The new AASM Scoring Manual recommends that sensors used for detection of respiratory effort are “either esophageal manometry, or calibrated or un-calibrated inductance plethysmography.” RIP is usually used in dual bands mode: RC and AB band respiration. That is the reason only two sensors are presented in our system. There are potentials that multiple RIP sensors might be used in wearable systems, xiphoid movement measurement for TCG calculation for example, our RIP module supporting multiple sensors has more advantages. RIP module supporting multiple inductive sensors described in this paper is an alternative solution for breathing monitoring systems.

### 3.5. Power Consumption of the RIP Module

The average current of the RIP module with two sensing belts under 3.3V DC supply condition is less than 7 mA, tested by the 34401A digital multimeter. Thus the total power consumption of the RIP module was less than 23.1 mW with an accuracy of ±15.51 μW. Compared with the large amount of excitation current (15 mA) for each sensor, system design with PAM and TDM can acquire a very good power consumption management.

In our RIP module, there is no need to design a high-stability LC oscillator. All sensors are excited by one constant-current source. As the current is fed to each sensor transiently, large feeding current is allowed for large amplitude response without increasing of overall power consumption. As described in this paper, the sensing unit is composed of a constant capacitor and the variable inductance belt to improve the AC impedance and hence the sensitivity. The proper selection of the capacitor to match the exciting high frequency current can be easily achieved by the help of an L/C meter. In Cohen's paper and the ‘LifeShirt’ patent [29], the TDM technique is also be mentioned. When a variable-frequency oscillator is used for inductance measurement, TDM is an effective method to eliminate frequency locking for multiple inductive bands used simultaneously. If an RIP system is designed with a variable-frequency oscillator for inductance measurement, the series tuned Clapps oscillator (a variation of the Colpitts oscillator), which will produce a high Q factor, is preferred for more accurate inductance measurement. As we mentioned, in this situation, design and implementation of a variable-frequency oscillator with high stability is still challenging.

The techniques of PAM and TDM can be used for other bio-medical sensors and systems aside from RIP. There are a series of bio-medical sensors that need to be excited by a constant voltage or current source such as NTC temperature sensor, RIP sensor, photoplethysmograph (PPG) probe sensor, and pressure sensor *etc.* PPG signal conditioning circuit, for example, is a typically embodiment of techniques of PAM and TDM. For sensors need large current or voltage excitation for high SNR, technique of PAM combining with TDM is desirable, especially for wearable systems.

## 4. Conclusions

We have developed an RIP module supporting multiple RIP sensors for wearable systems, and tested its performance on linearity, noninvasive ventilation measurement, average power consumption and real-time respiration measurement during movement. Linearity is an important quality for RIP, as output should change in proportionally both when the band is stretched and relaxed. Linearity tests with our self-made RIP sensor and testing apparatus shows that this RIP module can generate a good linearity with the cross-sectional area changing from 360 cm^2^ to 760 cm^2^. As for respiratory volume measurement, the comparison of breath-by-breath Vt measured from 15 healthy subjects demonstrated accurate lung volume estimation by this RIP module with two sensors connected and calibrated. Preliminary test of simulated airway obstruction showed that this new RIP can capture the obstructive respiratory event from the calibrated volume signal and paradoxical thoracic and abdominal movements. For real-time respiration measurement during movement, significant motion artifact can be inserted on RC and AB respiration by running movement. Adaptive filter with accelerometer signal as reference can be used to remove this kind of noise during movement. Though a large excitation current was used to for each RIP sensor to generate a large amplitude response, power consumption of this RIP module is actually very low. The PAM and TDM methods allow large feeding currents to each sensing unit without leading to high power consumption because of the low duty cycle. As MCU and integrated circuit chips were used in this RIP module, and the signal conditioning circuits are multiplexing for all sensing units, compact PCB and automatically gain setting for each sensing unit can be achieved. This RIP module can be used in wearable systems for RC and AB respiration monitoring.

## Figures and Tables

**Table 1.** Average Vt by RIP and PF-300 of 15 Volunteers and corresponding calibration coefficient K and M by QDC procedure.

**Subject**	**No. of breaths**	**Coefficient K**	**Coefficient M**	**Average Vt by RIP (mL)**	**Average Vt by PF-300 (mL)**	**Absolute error**
1	700	0.65	517.40	458 ± 69	461 ± 67	0.65%
2	647	1.47	351.04	489 ± 72	490 ± 60	0.20%
3	811	0.82	405.30	450 ± 67	456 ± 55	1.32%
4	864	0.76	503.20	412 ± 37	414 ± 34	0.48%
5	921	1.68	307.70	405 ± 57	410 ± 53	1.22%
6	858	0.78	379.40	442 ± 54	444 ± 40	0.45%
7	741	1.61	288.67	473 ± 82	480 ± 75	1.46%
8	1,073	0.91	478.11	406 ± 45	410 ± 37	0.98%
9	742	0.51	622.33	696 ± 72	713 ± 94	2.38%
10	915	0.99	318.00	391 ± 65	400 ± 75	2.25%
11	584	0.75	337.43	367 ± 59	362 ± 62	1.38%
12	519	0.64	320.86	460 ± 83	460 ± 113	0.00%
13	692	0.89	249.83	427 ± 72	422 ± 69	1.18%
14	952	1.04	156.07	363 ± 32	365 ± 37	0.55%
15	418	0.40	496.15	404 ± 112	402 ± 118	0.50%
